# Advancing syphilis diagnosis: multi-phase study evaluation of a TpN17-based double-antigen sandwich ELISA for detecting *Treponema pallidum* specific antibodies

**DOI:** 10.3389/fmicb.2025.1572785

**Published:** 2025-04-09

**Authors:** Ângelo Antônio Oliveira Silva, Larissa Carvalho Medrado Vasconcelos, Natália Erdens Maron Freitas, Talita Andrade Oliva, Miralba Freire Carvalho Ribeiro Silva, Isadora Cristina Siqueira, Edimilson Domingos Silva, Keila Gisele Azevedo Figueiredo Santos, Maria Amélia Virgens Lima, Nilson Ivo Tonin Zanchin, Fred Luciano Neves Santos

**Affiliations:** ^1^Advanced Public Health Laboratory, Gonçalo Moniz Institute, Oswaldo Cruz Foundation (Fiocruz-BA), Salvador, Brazil; ^2^Interdisciplinary Research Group in Biotechnology and Epidemiology of Infectious Diseases (GRUPIBE), Gonçalo Moniz Institute, Oswaldo Cruz Foundation (FIOCRUZ-BA), Salvador, Brazil; ^3^Medicine Course, Salvador University (UNIFACS), Salvador, Brazil; ^4^State Center for Diagnosis, Assistance, and Research (CEDAP), Bahia State Health Department (SESAB), Salvador, Brazil; ^5^Laboratory of Investigation in Global Health and Neglected Diseases, Gonçalo Moniz Institute, Oswaldo Cruz Foundation (FIOCRUZ-BA), Salvador, Brazil; ^6^Integrated Translational Program in Chagas Disease from Fiocruz (Fio-Chagas), Oswaldo Cruz Foundation (Fiocruz-RJ), Rio de Janeiro, Brazil; ^7^Diagnostic Technology Laboratory, Immunobiological Technology Institute (Bio-Manguinhos), Oswaldo Cruz Foundation (Fiocruz-RJ), Rio de Janeiro, Brazil; ^8^Structural Biology and Protein Engineering Laboratory, Carlos Chagas Institute, Oswaldo Cruz Foundation (Fiocruz-PR), Curitiba, Brazil

**Keywords:** syphilis, treponemal test, recombinant protein, TpN17, DAgS-ELISA

## Abstract

Syphilis, a sexually transmitted infection caused by the bacterium *Treponema pallidum*, has high incidence rates among adults, pregnant women, and newborns. Diagnostic procedures typically involve a treponemal test (such as ELISA, CMIA, and IFI), followed by a non-treponemal test (VDRL and RPR). This study aimed to assess the diagnostic performance of a double antigen sandwich ELISA (DAgS-ELISA) using the recombinant protein TpN17, analyzing serum samples from both infected and not infected with *T. pallidum*. A total of 712 samples were deemed eligible and recharacterized using VDRL, ELISA, and FTA-ABS, with 613 ultimately included in the evaluation: 180 *T. pallidum*-positive, 169 *T. pallidum*-negative, and 264 positive samples for other diseases. The assay was standardized using checkerboard titration and evaluated based on the area under the ROC curve (AUC), sensitivity, specificity, accuracy, likelihood values, diagnostic ratio, and Cohen’s Kappa index (*κ*). In phase I, positive and negative samples showed statistical differences (*p* < 0.0001) for the TpN17 protein. The ROC curve (AUC) was 98.7% and Cohen’s Kappa of 0.91, indicating almost perfect agreement with the reference tests. Phase II results demonstrated an AUC of 97.5%, specificity of 100%, sensitivity of 88.9%, accuracy of 94.3%, a positive likelihood ratio of 1.512, a negative likelihood ratio of 0.11, and a diagnostic odds ratio of 13,600, with a Cohen’s Kappa of 0.89. Cross-reactivity was observed in samples positive for Chagas disease (11.5%), HBV (2.6%), HCV (6.4%), and HTLV-1/2 (6.8%). Overall, TpN17 exhibited high diagnostic performance across all clinical stages of syphilis. Future research should expand the sample panel and explore new proteins to enhance DAgS-ELISA’s effectiveness and applicability for syphilis diagnosis across diverse clinical settings.

## 1 Introduction

Syphilis, a significant sexually transmitted infection (STI), is caused by the spirochete *Treponema pallidum* subspecies pallidum (*T. pallidum*) (Larsen et al., 1995; Singh and Romanowski, 1999). In the United States, syphilis ranks among the top three STIs in terms of reported case rates, experiencing a fivefold increase in the incidence of primary and secondary syphilis since 2000 (Hazra et al., 2022). In Brazil, between 2001 and 2017, 188,630 cases of congenital syphilis (CS) and 235,895 cases of syphilis in pregnancy (SiP) were reported, with approximately all microregions documenting at least one case. Between 2012 and 2016, the incidence of CS rose sharply across most Brazilian states, particularly in the South, Southeast, and Central-West regions, while the relative risk for SiP increased by approximately 400%, highlighting the growing challenge of syphilis control (Silva et al., 2022). Given the increasing number of syphilis cases in Brazil, the reliance on rapid tests for diagnosing and reporting cases presents certain challenges. As highly sensitive screening methods are essential, the development and integration of new diagnostic approaches—such as computational tools (Albuquerque et al., 2023), voltammetry-based assays (Barros et al., 2022), and serological innovations—should be considered for updating the diagnostic algorithm.

The clinical progression of syphilis unfolds in distinct stages. Primary syphilis presents with a painless, indurated chancre at the site of bacterial inoculation and replication (Chapel et al., 1977; Chapel, 1978). This lesion, typically with a clean base, signifies the initial infection. Within approximately 8 weeks, untreated cases progress to secondary syphilis, characterized by diverse cutaneous manifestations, including macular to maculopapular rashes, follicular eruptions, and, in some cases, pustular or nonpruritic lesions, often affecting the palms and soles (Singh and Romanowski, 1999; Chapel, 1980). If untreated, the disease enters a latent or asymptomatic phase, which may ultimately lead to tertiary syphilis, a stage associated with severe systemic complications, including cardiovascular disease and neurosyphilis, which can present asymptomatically or with meningovascular involvement (Larsen et al., 1995; Singh and Romanowski, 1999). Additionally, vertical transmission during pregnancy can result in congenital syphilis, leading to multi-organ involvement in neonates, who may present with or without clinical symptoms (Singh and Romanowski, 1999; Blank et al., 1977; Wendel, 1988).

Syphilis diagnosis relies on a combination of non-treponemal (NTT) and treponemal tests (TT). NTTs, such as the Venereal Disease Research Laboratory (VDRL) test, are used to confirm positive TT and monitor treatment response, detecting anticardiolipin antibodies that typically emerge within 1–2 weeks after chancre development (Larsen et al., 1995; Singh and Romanowski, 1999). TTs, which are employed for screening and confirmatory diagnosis, include enzyme-linked immunosorbent assays (ELISA), rapid diagnostic tests (RDT), chemiluminescence immunoassays (CMIA), and indirect immunofluorescence tests (IIFT). These assays qualitatively detect IgG or IgM antibodies against *T. pallidum* (Larsen et al., 1995; Singh and Romanowski, 1999; Clyne and Jerrard, 2000; Forrestel et al., 2020). Furthermore, some of these tests detect both IgG and IgM simultaneously and can be semi-quantitative (IIFT) or even quantitative (CMIA). However, quantification is not relevant for the diagnosis or monitoring of the infection, as is the case with NTTs. While TTs generally exhibit superior diagnostic accuracy compared to NTTs due to their specificity for pathogen-derived antibodies (Park et al., 2019), their performance is influenced by the antigenic composition of the assays, affecting sensitivity and specificity (Larsen et al., 1995; Liu et al., 2019).

Previous studies conducted by our group demonstrated that two recombinant *T. pallidum* proteins, TpN17 and TmpA, yielded high sensitivity, specificity, and accuracy (>90%) when used in indirect ELISA (Silva et al., 2020). Notably, TpN17 exhibited a diagnostic odds ratio 36.4 times greater than TmpA (Silva et al., 2024). Building upon these findings, we sought to evaluate the diagnostic performance of TpN17 using the double-antigen sandwich ELISA (DAgS-ELISA) platform. DAgS-ELISA has been recognized for its high sensitivity enabling the simultaneous detection of IgG and IgM antibodies (Sas et al., 2018). Its design mitigates issues commonly encountered in indirect ELISA, such as false positives and undetectable IgM levels. Therefore, DAgS-ELISA represents a promising alternative for enhancing the specificity and sensitivity of syphilis serodiagnostics (He et al., 2011). This study aims to assess the diagnostic performance of DAgS-ELISA incorporating the TpN17 recombinant protein, with the potential to improve diagnostic accuracy and strengthen syphilis surveillance and control efforts.

## 2 Materials and methods

### 2.1 Recombinant proteins synthesis

The recombinant *T. pallidum* protein TpN17 was synthesized following the methodology described by Silva et al. (2020). A synthetic gene encoding the TpN17 protein from the *T. pallidum* Nichols strain was obtained from GenScript (Piscataway, NJ, United States) and subcloned into the pET28a expression vector. This construct was transformed into *Escherichia coli* BL21-Star (DE3) for protein expression (Hanahan, 1983). Bacterial cultures were grown in Luria-Bertani broth supplemented with 50 μg/mL kanamycin at 37°C for 16 h. The cultures were subsequently diluted (1:20) in fresh medium and incubated until reaching an optical density (OD) of 0.6–0.8 at 600 nm. Protein expression was induced using 500 μM isopropyl β-D-1-thiogalactopyranoside (IPTG) for 4 h at 37°C. Cell disruption was performed using either a microfluidizer (Microfluidics Model M-110 L, Hyland Scientific, Stanwood, WA, United States) or chemical lysis. The recombinant protein was purified via affinity and ion exchange chromatography. Protein concentration was measured using the Qubit 12.0 fluorometric assay (Invitrogen Technologies, Carlsbad, CA, United States), and purity was confirmed by SDS-PAGE with Coomassie Brilliant Blue G-250 staining (Laemmli, 1970), ensuring compliance with experimental standards.

### 2.2 Peroxidase labeling process

The horseradish peroxidase (HRP)-labeled TpN17 conjugate (TpN17-HRP, Lot: 21OEXCJHO19Z) was provided by the Laboratory of Serological Tests (Bio-Manguinhos, FIOCRUZ-RJ). The conjugation process was performed via oxidative amination using sodium periodate (Freitas et al., 2022). Following enzyme-protein binding, sodium borohydride was added to stabilize the reaction (Tijssen and Kurstak, 1984). While the exact conjugation protocols remain proprietary due to industrial confidentiality policies, the process was adapted from established methodologies in the literature (Nakane and Kawaoi, 1974; Farr and Nakane, 1981; Tijssen and Kurstak, 1984; Pavliuchenko et al., 2019).

### 2.3 Sample collection

This study was conducted in two phases to evaluate the diagnostic performance of TpN17 in a DAgS-ELISA for syphilis. Phase I assessed the ability of TpN17 to distinguish between *T. pallidum*-positive (TpP) and syphilis-negative (TpN) samples. Sampling was based on convenience, without predefined group sizes. A total of 100 TpP samples were collected from the State Center Specialized in Diagnosis, Assistance, and Research (CEDAP), and 169 TpN samples were obtained from the Foundation of Hematology and Hemotherapy of the State of Bahia (HEMOBA Foundation). The TpP samples included 20 primary syphilis cases, 39 secondary syphilis cases, and 41 early latent syphilis cases, all confirmed through serological testing (rapid immunochromatographic test and VDRL) and clinical examinations at a reference center.

**Figure 1:**
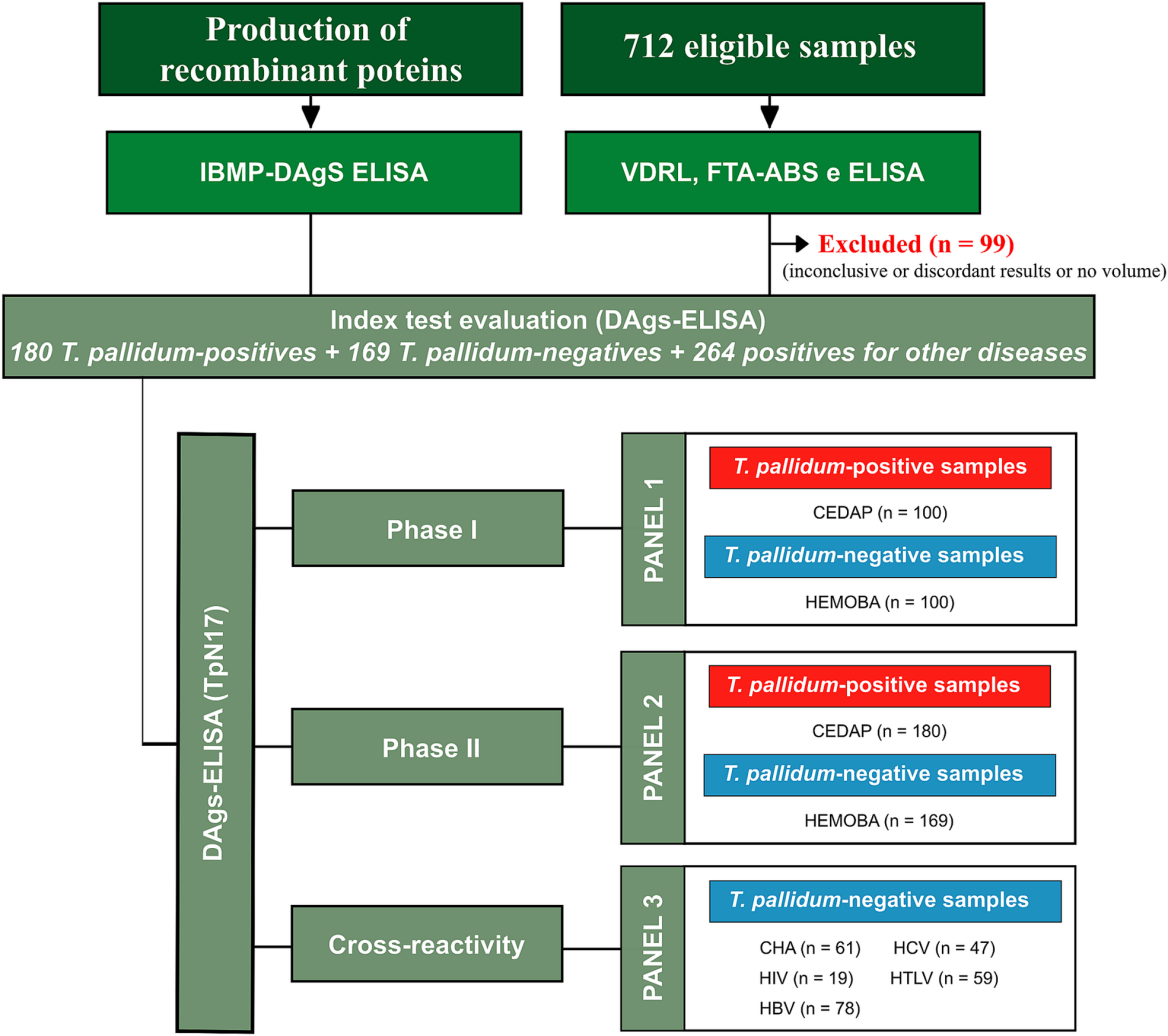
Flowchart of the diagnostic performance assessment of DAgS-ELISA using recombinant antigen TpN17 for serological diagnosis of syphilis. Reference Standards Tests: VDRL (Venereal Disease Research Laboratory); ELISA (enzyme-linked immunosorbent assay); IIFT (indirect immunofluorescence - IgG); DAgS-ELISA (Double-antigen Sandwich ELISA); CHA (chronic Chagas disease); HBV (hepatitis B); HCV (hepatitis C); HIV (human immunodeficiency virus); HTLV (human T cell lymphotropic virus).

Phase II aimed to determine diagnostic parameters such as sensitivity, specificity, and accuracy. Sample size was calculated using OpenEpi open-source software^1^ (Dean et al., 2013), assuming 99% sensitivity and specificity with a 95% confidence interval (CI) and an absolute error of 1.5%. The calculation determined a minimum required sample size of 338 (169 TpP and 169 TpN). From the 712 initially eligible samples, 99 were excluded due to insufficient volume or discordant/inconclusive results, leaving 613 samples for analysis (Figure 1). The TpP samples for this phase included 30 primary syphilis cases, 77 secondary syphilis cases, and 73 early latent syphilis cases. An additional 264 samples from individuals with unrelated diseases, including chronic Chagas disease, hepatitis B, hepatitis C, HIV-1/2, and HTLV-1/2, were included to assess cross-reactivity and were sourced from the HEMOBA Foundation.

All serum samples underwent retesting for *T. pallidum* antibodies using a VDRL test kit (Wiener lab., Rosario, Argentina) for non-treponemal testing and two treponemal assays: the anti-*Treponema pallidum* IIFT (IgG) test (Euroimmun Medizinische Labordiagnostika AG, Lübeck, Germany) and the recombinant ELISA v.4.0 test (IgG) (Wiener lab., Rosario, Argentina). Samples yielding discordant or inconclusive results were excluded from the analysis. Each sample was assigned a unique identifier to ensure a blinded analysis.

### 2.4 Double-antigen sandwich ELISA optimization and procedure

The DAgS-ELISA assay was optimized using checkerboard titration method to determine the optimal concentrations of antigens, serum, and antigen–enzyme conjugate (HRP). TpN17 was diluted in 0.05 M carbonate–bicarbonate buffer (pH 9) at final concentrations ranging from 3.125 to 100 ng per well and applied to 96-well high-binding microplates (Microlon 600 half-area, Greiner Bio-One, Kremsmünster, Austria). After a 15-min incubation at room temperature, microplates were blocked with 50 μL per well of WellChampion^®^ synthetic blocking buffer (Kem-En-Tec, Taastrup, Denmark) for 15 min. The plates were then dried at 37°C for 90 min and washed with PBS containing 0.05% Tween-20 (PBS-T). Serum samples were tested in duplicates, both undiluted and at serial dilutions from 1:2 to 1:32 in PBS-T, followed by incubation at 37°C for 30 min. After washing, 50 μL of HRP-labeled antigen was added at dilutions ranging from 1:1000 to 1:32000, followed by incubation at 37°C for 30 min. The plates were then washed, and 50 μL of tetramethylbenzidine (TMB) substrate (Kem-En-Tec Diagnostics A/S, Taastrup, Denmark) was added to initiate the enzymatic reaction, which proceeded for 10 min at room temperature in the dark. The reaction was stopped by adding 25 μL of 0.2 M sulfuric acid (H₂SO₄), and absorbance was measured at 450 nm using a microplate spectrophotometer (SPECTRAMAX 340PC^®^, San José, CA, United States).

### 2.5 Statistical analysis

Statistical analyses were conducted using GraphPad Prism v9.5.1 (GraphPad Software, San Diego, CA, United States). Variables were analyzed using arithmetic and geometric means, standard deviation (SD), and coefficients of variation. Geometric means were reported with 95% confidence interval (CI). Data normality was assessed using the Shapiro–Wilk test. For datasets that followed a normal distribution, the Student’s t-test was applied for comparative analysis. For normally distributed datasets, comparisons were performed using Student’s *t*-test, whereas non-normally distributed data were analyzed using the Wilcoxon-Mann–Whitney or Kruskal-Wallis tests. Statistical significance was set at *p* < 0.05.

Cut-off (CO) values were established based on the highest area under the receiver operating characteristic (ROC) curve (AUC). The reactivity index (RI) was calculated as the ratio of the optical density (OD) to the CO. Samples were classified as positive if RI ≥ 1.0, negative if RI < 1.0, and inconclusive (gray zone) if within ±10% of 1.0. Assay accuracy was determined based on AUC classification: low (0.51–0.61), moderate (0.62–0.81), high (0.82–0.99), or outstanding (1.0) (Swets, 1988). Performance parameters, including sensitivity (Sen), specificity (Spe), accuracy (Acc), likelihood ratios (LR+ and LR-), and diagnostic odds ratio (DOR), were evaluated. Agreement between TpN17-DAgS-ELISA and reference tests was assessed using Cohen’s Kappa (*κ*), with interpretations as follows: 1.0 ≤ κ ≥ 0.81 (almost perfect agreement), 0.80 ≤ κ ≥ 0.61 (substantial agreement), 0.60 ≤ κ ≥ 0.41 (moderate agreement), 0.40 ≤ κ ≥ 0.21 (fair agreement), 0.20 ≤ κ ≥ 0 (slight agreement), or κ = 0 (poor agreement) (Landis and Koch, 1977).

The study adhered to the Standards for the Reporting of Diagnostic Accuracy Studies (STARD) guidelines, with a detailed checklist included in Supplementary Table S1 to ensure comprehensive reporting and transparency.

## 3 Results

A total of 180 previously collected and anonymized serum samples from individuals who tested positive for *T. pallidum* (TpP) were included in phase studies I and II. The median age in the TpP group was 29 years, with an interquartile range of 13 years. A significant gender disparity was observed, with a female-to-male ratio of 1:8.47. Regarding sexual orientation, 4.4% (8/180) of participants identified as bisexual, 30.5% (55/180) as heterosexual, 60% (108/180) as homosexual, and 5% (9/180) did not specify their orientation. Clinically, the distribution across syphilis stages varied, wih 15.5% (28/180) classified as primary syphilis, 41.1% (74/180) as secondary syphilis, and 43.3% (78/180) as recent latent syphilis, demonstrating the diverse clinical presentation of the infection. The *T. pallidum*- negative (TpN) group consisted of 169 individuals, with a more balanced female-to-male ratio of 1:1.45. All individuals in this group were residents of Bahia. However, the absence of age-related data for this group limited direct demographic comparisons between the two study populations.

**Figure 2:**
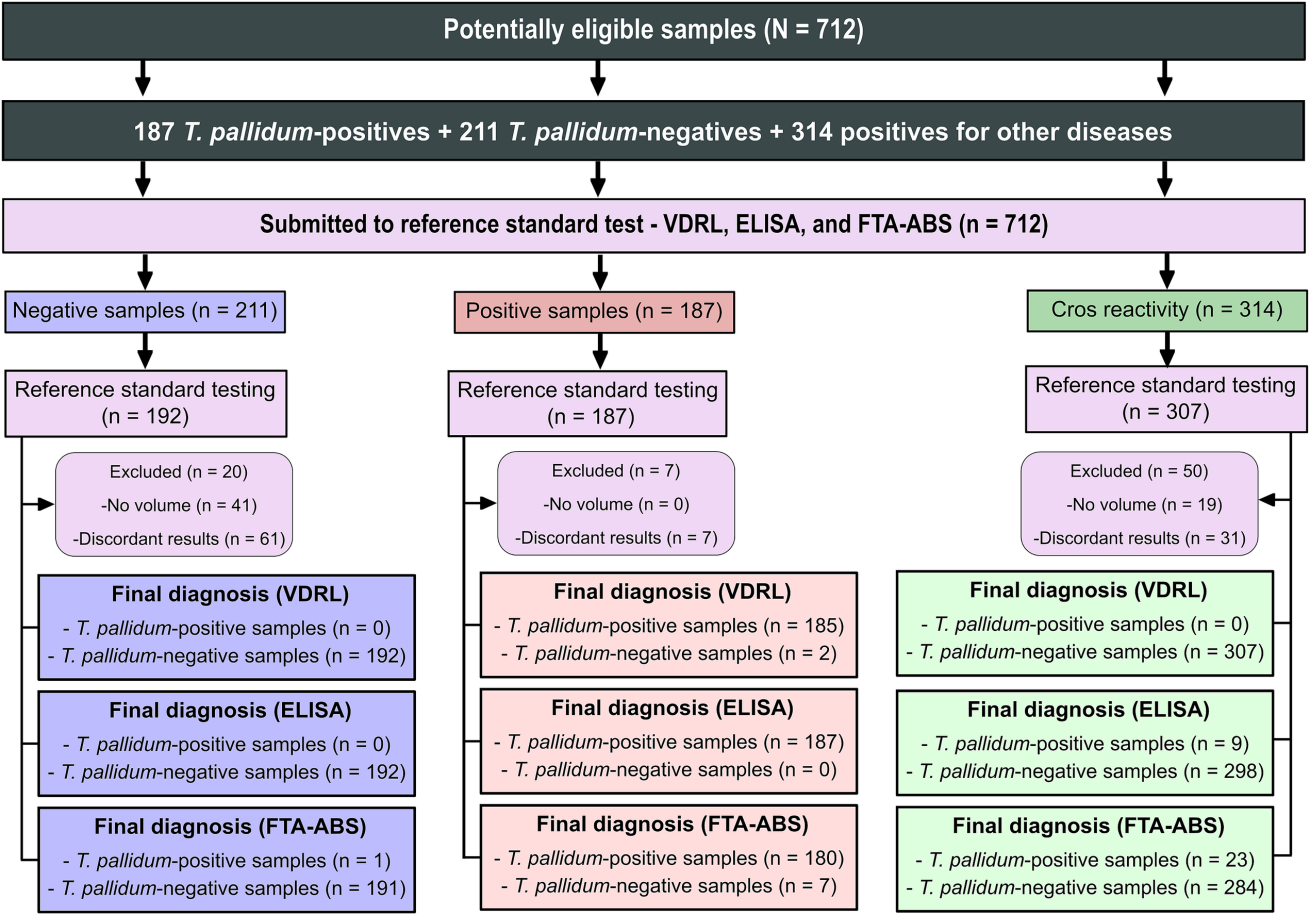
Sera characterization from *Treponema pallidum*-positive and negative samples tested with VDRL, ELISA, and IIFT. Key diagnostic tests are abbreviated as follows: VDRL (Venereal Disease Research Laboratory); ELISA (enzyme-linked immunosorbent assay); IIFT (indirect immunofluorescence - IgG).

A total of 712 serum samples were analyzed to determine their serological status for *T. pallidum* infection (Figure 2). Initial screening was performed using both non-treponemal (VDRL) and treponemal tests (ELISA and FTA-ABS) to detect non-specific and specific antibodies, respectively. Of these, 99 samples (13.9%) were excluded due to discordant or inconclusive results, or insufficient volume. Among the remaining samples, 180 were initially classified as syphilis-positive, but seven were excluded due to inconsistencies in testing: all seven were reactive in ELISA, two were non- reactive in VDRL, and all seven were reactive in IIFT. In the TpN group, 169 samples were confirmed as negative. However, one sample tested positive in IIFT, and 41 were excluded due to insufficient volume. In the group of individuals with unrelated diseases, 264 samples tested negative for syphilis, indicating no significant cross-reactivity or syphilis co-infections. However, 31 samples yielded discordant results, with nine reactive in ELISA and 23 in IIFT, suggesting potential false positives or underlying conditions affecting the test performance. Additionally, 19 samples were excluded due to insufficient volume, further limiting the analysis.

Optimization of the DAgS-ELISA assay was performed using checkerboard titration to determine the optimal dilutions for antigen coating, antigen–enzyme conjugate (HRP), and serum samples. Selection criteria prioritized maximizing the signal-to-noise ratio (SNR) while maintaining OD thresholds below 0.25 for negative samples and above 1.00 for positive samples. Based on these criteria, the TpN17 antigen concentration was set at 50 ng, with HRP-labeled antigen and serum dilutions optimized at 1:4000 and 1:4, respectively.

**Figure 3:**
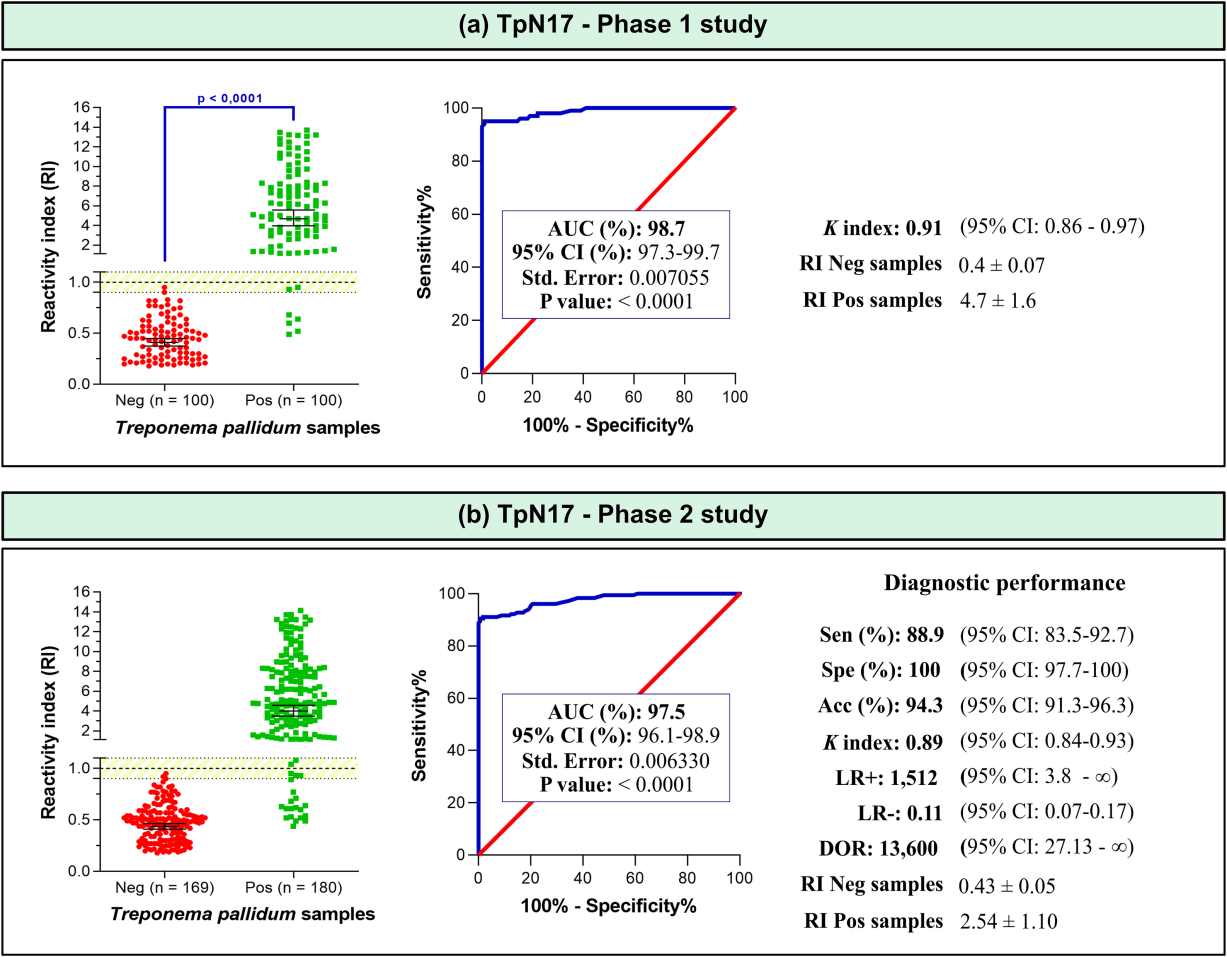
The reactivity index and diagnostic performance parameters of TpN17 for *Treponema pallidum*-positive and negative serum samples. The cut-off is established at a reactivity index value of 1.0, with the shaded area indicating the gray zone (RI = 1.0 ± 0.10). Horizontal lines and accompanying numbers illustrate the geometric means (±95% CI) for each result group. Key diagnostic metrics are abbreviated as follows: AUC (Area Under Curve); Sen (Sensitivity); Spe (Specificity); Acc (Accuracy); LR+ (positive likelihood ratio); LR- (negative likelihood ratio); DOR (Diagnostic Odds ratio). Labels Neg and Pos denote negative and positive samples, respectively.

In phase 1 study, which included 100 positive and 100 negative samples, the TpN17 antigen exhibited a high discriminatory capacity, achieving an AUC value of 98.7%. The Cohen’s Kappa index was 0.91, indicating near-perfect agreement with reference tests (Figure 3A; Supplementary Table S2). In the phase 2 study, which included 180 positive and 169 negative samples, TpN17 maintained strong diagnostic performance with an AUC of 97.5%. Sensitivity was 88.9%, specificity was 100%, and accuracy was 94.3%. Despite the reduced sensitivity, the Cohen’s Kappa index remained high at 0.89, confirming near-perfect agreement. Additionally, the test yielded a positive LR ratio of 1,512, a negative LR of 0.11, and a DOR of 13,600 (Figure 3B; Supplementary Table S2).

Within the defined inconclusive range (RI = 1.0 ± 10%), a small proportion of *T. pallidum*-positive samples produced inconclusive results, with four (4%) in phase 1 and seven (4%) in phase 2. The mean reactivity index (RI) for *T. pallidum*-positive samples was 5.0 ± 2.1 in phase 1 and 3.9 ± 2.5 in phase 2. For negative samples, RI values remained consistent at 0.4 ± 1.5 across both phases.

**Figure 4:**
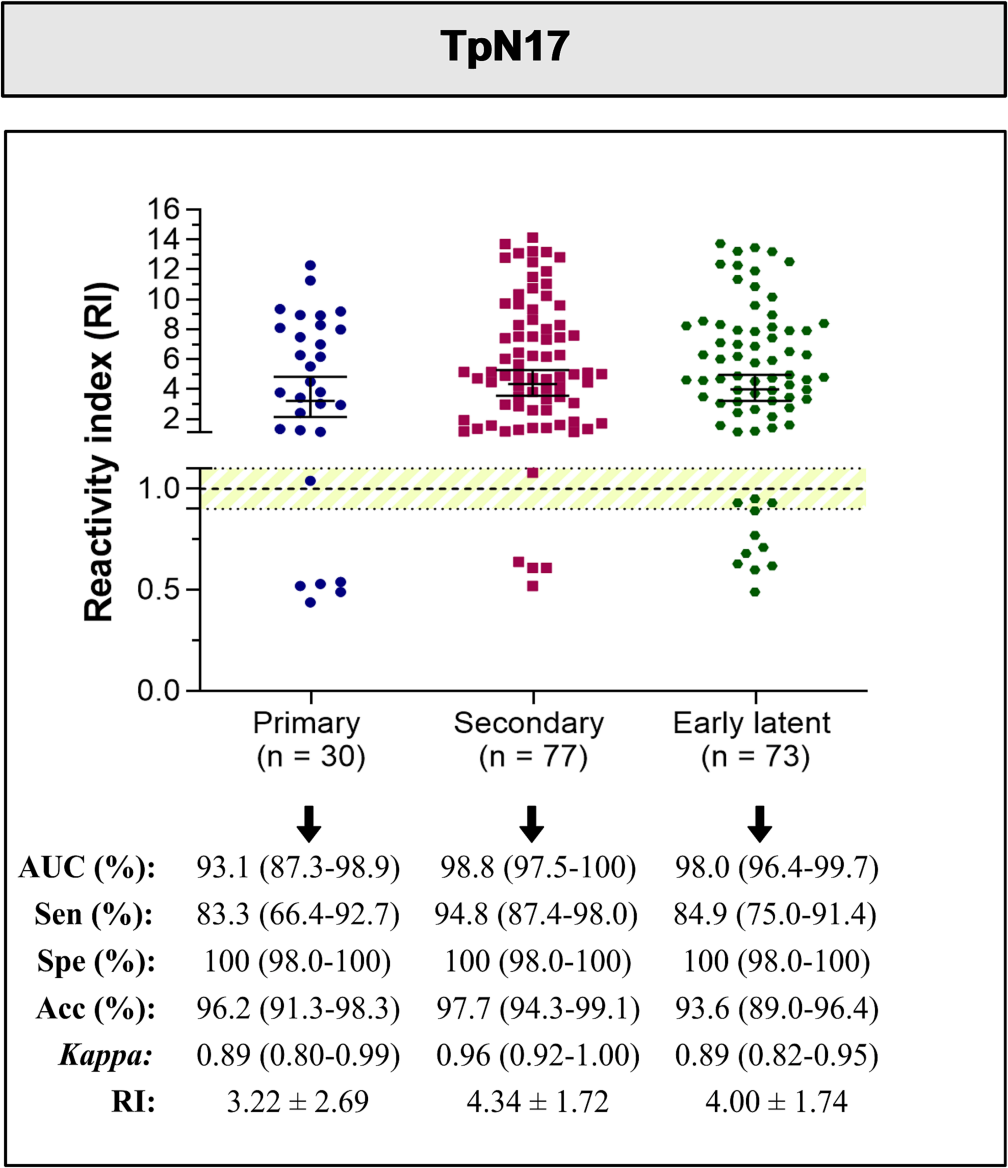
The reactivity index and diagnostic performance parameters of the TpN17, stratified by clinical stage, for *Treponema pallidum*-positive serum samples. The cut-off is established at a reactivity index value of 1.0, with the shaded area indicating the gray zone (RI = 1.0 ± 0.10). Horizontal lines and accompanying numbers illustrate the geometric means (±95% CI) for each result group. Key diagnostic metrics are abbreviated as follows: AUC (Area Under Curve); Sen (Sensitivity); Spe (Specificity); Acc (Accuracy).

When assessing the performance of the TpN17 antigen across different clinical stages of syphilis, the AUC remained above 93.1% for primary, secondary, and early latent syphilis (Figure 4). Sensitivity was highest for secondary syphilis at 98.8%, followed by early latent syphilis at 98.0% and primary syphilis at 93.1%. Specificity remained consistently high at 100% across all stages. Accuracy ranged between 93.6 and 97.7%. Cohen’s Kappa analysis demonstrated almost perfect agreement, with values between 0.89 to 0.96. Mean RI values for *T. pallidum*-positive samples were 3.22 ± 2.69 for primary syphilis, 4.34 ± 1.72 for secondary syphilis, and 4.00 ± 1.74 for early latent syphilis.

**Figure 5:**
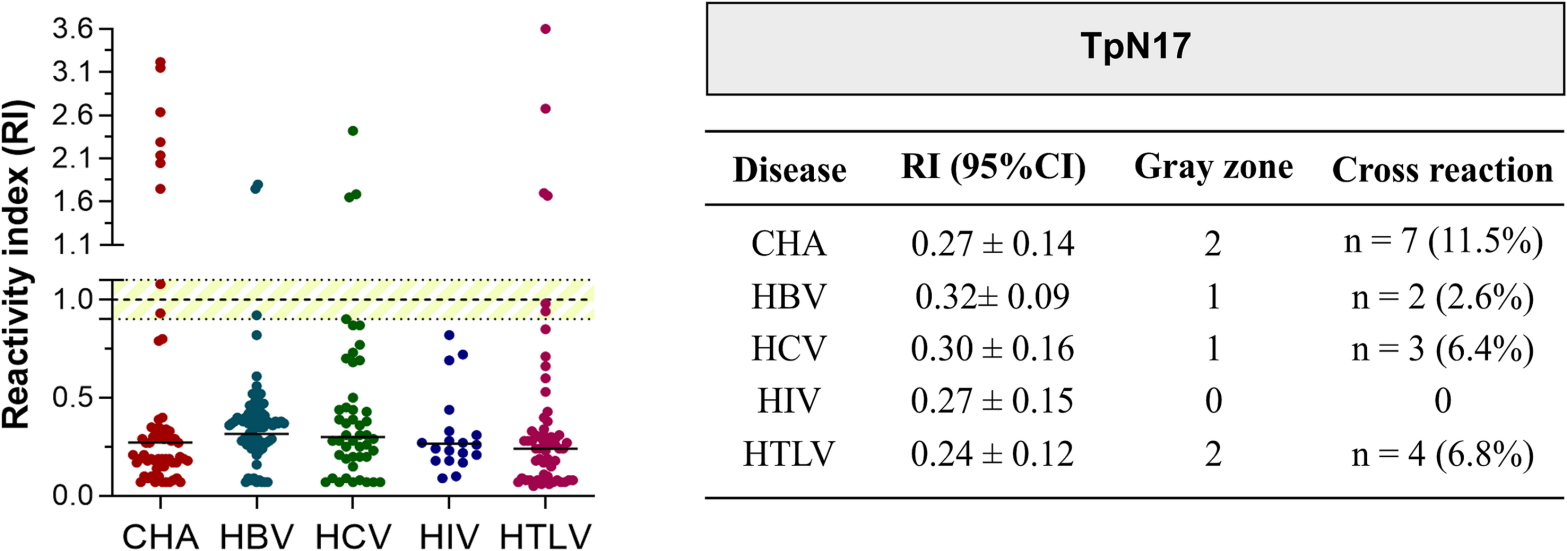
Analysis of the cross-reactivity of the *Treponema pallidum*-recombinant proteins with sera from various infectious parasitic and viral diseases. The cut-off value is reactivity index = 1.0 and the shaded area represents the gray zone (RI = 1.0 ± 0.10). CHA (chronic Chagas disease); HBV (hepatitis B); HCV (hepatitis C); HIV (human immunodeficiency virus); HTLV (human T cell lymphotropic virus).

To assess potential cross-reactivity, 264 serum samples from individuals with unrelated infectious diseases were analyzed (Figures 1, 2). A total of 16 cases of cross-reactivity were observed, including seven from *Trypanosoma cruzi*-positive samples (1.54%), two from hepatitis B-positive samples (2.6%), three from hepatitis C-positive samples (6.4%), and four from HTLV-1/2-positive samples (6.8%). In addition, six samples fell within the designated gray zone, including two *T. cruzi*-positive samples (3.3%), one hepatitis B virus (HBV)-positive sample (1.3%), one hepatitis C virus (HCV)-positive sample (2.1%), and two HTLV-1/2-positive samples (3.4%). Among the cross-reactive samples, the highest RI value was observed for HBV (0.32 ± 0.09), while the lowest RI value was recorded for HTLV-1/2 (0.24 ± 0.12) (Figure 5; Supplementary Table S3).

## 4 Discussion

This study evaluated the diagnostic performance of the TpN17 antigen using the DAgS-ELISA platform for detecting anti-*T. pallidum* antibodies. The assays demonstrated high diagnostic efficacy, achieving AUC values exceeding 97% in both phase 1 and 2 studies, underscoring its excellent ability to distinguish between positive and negative samples. These findings align with previous studies using indirect ELISA (Silva et al., 2024) and suggest that TpN17 provides superior discrimination compared to other recombinant proteins and *T. pallidum* cell lysates reported in the literature (Gerber et al., 1996; Fujimura et al., 1997; Silva et al., 2020; Silva et al., 2024). Furthermore, TpN17-based assays produced minimal inconclusive results across all syphilis stages and exhibited low cross-reactivity with other infectious diseases, highlighting their specificity and potential utility in clinical diagnostics.

The initial phase of diagnostic assay development focused on optimizing assay components to achieve maximum sensitivity and specificity. In our study, optimization involved determining the ideal antigen concentration, HRP-labeled antigen titer, and serum dilution. The highest SNR was achieved using 50 ng of TpN17 per well, a 1:4000 dilution of the HRP conjugate in PBS, and a 1:4 serum dilution in PBS-Tween. Although no direct comparisons with DAgS-ELISA for syphilis were found, studies on autoimmune and other infectious diseases have used various optimization conditions. Our study adopted a protocol that utilized a lower antigen concentration and a shorter incubation period compared to the conventional overnight process, simplifying the blocking step while maintaining high assay efficiency. Reported antigen concentrations for microplate sensitization vary widely in the literature, ranging from 25 ng/μL (Freitas et al., 2022) to 800 ng/μL (Jiao et al., 2012). Intermediate concentrations have also been reported, including 40 ng/well (Chen et al., 2005), 60 ng/μL (Hu et al., 2008), 100 ng/well (Liu et al., 2016), 400 ng/μL (Li et al., 2010; Guo et al., 2022), and 500 ng/μL (Li et al., 2014). The choice of sensitization buffer varied across studies, with PBS (Li et al., 2014), TRIS HCl (Li et al., 2010), and carbonate buffers being commonly used (Chen et al., 2005; Hu et al., 2008; Jiao et al., 2012; Freitas et al., 2022; Guo et al., 2022), typically applied overnight at 4°C to enhance antigen binding (Chen et al., 2005; Hu et al., 2008; Li et al., 2010; Li et al., 2014; Liu et al., 2016; Guo et al., 2022). Blocking strategies also differed significantly among studies, employing agents such as newborn calf serum (Li et al., 2014), PBS-Tween with skimmed milk powder (Guo et al., 2022), bovine serum albumin (Chen et al., 2005), and a combination of sucrose, Na-casein, and bovine serum albumin in PBS (Liu et al., 2016). Some studies used skimmed milk powder in PBS (Jiao et al., 2012), while Freitas et al. (2022) uniquely employed the WellChampion reagent as a sensitizer, blocker, and stabilizer, demonstrating an alternative approach to optimizing ELISA conditions.

In our assay standardization, a uniform sensitization buffer and a shorter incubation time were adopted, significantly streamlining the process. The decision to use a 1:4 serum dilution in PBS-Tween balanced optimal sensitivity and specificity, in contrast to other studies that varied dilution ratios between 1:1 and 1:20 (Jiao et al., 2012; Liu et al., 2016; Freitas et al., 2022; Guo et al., 2022). Similarly, conjugated protein concentrations varied widely in previous studies, ranging from 1:500 to 1:2000 (Hu et al., 2008; Li et al., 2010; Jiao et al., 2012; Freitas et al., 2022). Our approach, using a 1:4000 HRP-conjugated TpN17 dilution, reflects a tailored optimization strategy that enhances assay performance while maintaining high specificity and sensitivity, crucial for reliable clinical diagnostics.

In both phase 1 and 2 studies, TpN17 exhibited outstanding diagnostic performance, with AUC values of 98.5 and 97.5%, respectively. In phase 2 study, the assay demonstrated a sensitivity of 88.9%, specificity of 100%, and accuracy of 94.3%, with a Cohen’s Kappa index of 0.89, indicating near-perfect agreement with reference tests. The diagnostic efficacy of TpN17 was further highlighted by its likelihood ratios and the diagnostic odds ratio. The LR+ of 1,512 strongly increases the probability of correctly identifying syphilis-positive individuals, while the LR- of 0.11 demonstrated a moderate reduction in the likelihood of false negatives. The DOR of 13,600 emphasized the high discriminative power of TpN17, making it 13,600 times more likely to yield a positive result in syphilis-positive individuals compared to uninfected individuals.

The findings of this study align closely with those reported by Freitas et al. (2022), which assessed *Trypanosoma cruzi* chimeric antigens for diagnosing Chagas disease. The AUC values reported in that study ranged from 97.5 to 99.5%, comparable to the 97.5 to 98.5% observed for TpN17 in our Phase 1 and 2 analyses. However, the sensitivity of the *T. cruzi* antigens, ranging from 74.4 to 88.4%, was slightly lower than that of TpN17, which exhibited a sensitivity of 88.9%. Despite this difference, both assays demonstrated high specificities, ranging from 96.6 to 100%. The accuracy of *T. cruzi* antigens, which varied between 87.1 and 93.4%, was also similar to the 94.3% accuracy observed for TpN17, highlighting the effectiveness of the DAgS-ELISA platform for detecting infectious diseases.

Further comparative analysis revealed notable differences in assay performance across various pathogens. In the context of viral infections, Qin et al. (2020) evaluated three commercial ELISA kits for hepatitis C and reported that DAgS-ELISA yielded a slightly lower positivity rate than indirect ELISA, a trend also observed in our phase 2 study. Similarly, Hu et al. (2008) found that DAgS-ELISA exhibited high specificity and positivity rates for diagnosing hepatitis E virus, confirming its consistency with reference ELISAs. Conversely, Li et al. (2010) demonstrated that DAgS-ELISA achieved higher sensitivity in detecting hepatitis B compared to the reference test (Architect CMIA), emphasizing assay performance variability across diseases. Qin et al. (2020) suggested that the increased sensitivity of DAgS-ELISA might stem from its ability to detect antibodies regardless of immunoglobulin class. However, limitations in indirect ELISA, such as false-positive results and the inability to detect low IgM levels (He et al., 2011), highlight the nuanced differences between these assay formats. While DAgS-ELISA has shown superior specificity and sensitivity in certain studies, variability in performance underscores the need for continued optimization and validation to ensure its reliability in diverse clinical settings.

Guo et al. (2022) demonstrated that DAgS-ELISA exhibited exceptional diagnostic accuracy for Chikungunya, with a sensitivity of 100%, specificity of 99.5%, accuracy of 99.6%, and a Cohen’s Kappa index of 0.98, indicating near-perfect agreement with the reference test. However, their results were influenced by the timing of sample collection, with sensitivity declining during the early phase of infection when PCR was used as the reference. In contrast, TpN17-DAgS-ELISA maintained consistent performance across different stages of syphilis, demonstrating a significant diagnostic advantage. A study on COVID-19 using DAgS-ELISA reported a positivity rate of 70% (Chen et al., 2005). However, this study included only 18 positive cases, 20 negative cases, and a serially diluted control, making its findings less robust. In contrast, our study utilized a more extensive serological panel and found that TpN17-DAgS-ELISA exhibited superior sensitivity compared to the COVID-19 assay. Discrepancies in results may be attributed to misclassification of SARS-CoV-2 samples in earlier studies, a limitation we addressed by reanalyzing all samples using three distinct commercial tests to ensure accuracy and assay robustness.

Liu et al. (2016) compared indirect ELISA and DAgS-ELISA for detecting Varicella-Zoster virus and found that while both assays effectively differentiated between positive and negative samples, indirect ELISA demonstrated superior sensitivity. Similarly, in our study, TpN17-DAgS-ELISA exhibited slightly lower sensitivity than indirect ELISA but maintained high overall diagnostic performance. In comparison to the FAMA test (fluorescent antibody to membrane antigen), DAgS-ELISA achieved a sensitivity of 95.1%, a specificity of 100%, and a concordance rate of 97.6%. The ability of DAgS-ELISA to detect total antibodies (IgG, IgM, and IgA) enhances its diagnostic utility, particularly for identifying weakly positive samples (Liu et al., 2016; Qin et al., 2020). Jiao et al. (2012) reported that DAgS-ELISA achieved nearly 100% sensitivity and specificity in detecting severe fever with thrombocytopenia syndrome virus. Unlike indirect ELISA, which detects class-specific antibodies, DAgS-ELISA provides a broader detection spectrum. However, a key limitation of DAgS-ELISA is its inability to distinguish between serological cure and reinfection, which may be critical in clinical contexts requiring differentiation between past exposure and active infection. Despite this, its broad-spectrum detection enhances its applicability in comprehensive serological evaluations, although confirmatory testing may be necessary in specific cases.

In our cross-reactivity analysis, we assessed 264 samples positive for Chagas disease, HBV, HCV, HIV-1/2, and HTLV-1/2. Cross-reactivity was observed in seven *T. cruzi*-positive samples, two HBV samples, three HCV samples, and four HTLV-1/2 samples. To rule out false positives due to anti-*T. pallidum* IgG, these samples were further tested with VDRL, ELISA, and FTA-ABS, yet the reactivity rates remained higher than expected. In a phase 1 study, indirect ELISA demonstrated no cross-reactivity with non-bacterial diseases, whereas an 8.6% cross-reactivity rate was observed for TpN17 with leptospirosis samples (Silva et al., 2024). However, in phase 2, cross-reactivity was minimal, with only one *T. cruzi*-positive sample yielding a false-positive result, suggesting that modifications in assay conditions or antigen preparation can effectively reduce cross-reactivity.

Additional comparative insights come from Guo et al. (2022), who evaluated cross-reactivity of DAgS-ELISA for Chikungunya virus against hantavirus, hemorrhagic fever with renal syndrome, dengue, and bunyavirus samples. They found cross-reactivity rates of 0.5% for DAgS-ELISA and 2.3% for indirect ELISA (IgG). Similarly, Hu et al. (2008) assessed a new DAgS-ELISA for hepatitis E virus and found a 2% cross-reactivity rate, primarily with hepatitis C samples. Freitas et al. (2022) analyzed cross-reactivity in a DAgS-ELISA for Chagas disease and found no false positives, highlighting its specificity. Differences in cross-reactivity among studies may be attributed to the choice of antigenic matrix, with recombinant antigens (Hu et al., 2008; Guo et al., 2022) exhibiting higher cross-reactivity than chimeric recombinant antigens (Freitas et al., 2022). Variations in cut-off values and optical density thresholds between DAgS-ELISA and indirect ELISA may also significantly impact results. Ge et al. (2012) proposed that even if nonspecific antibodies bind in the solid phase, they should not bind to the conjugated antigen in the liquid phase, theoretically resulting in a low background signal. This principle is crucial for minimizing false positives and enhancing the specificity of serological tests.

## 5 Conclusion

The TpN17 antigen demonstrated high diagnostic performance in distinguishing between syphilis-positive and negative samples, though performance metrics slightly declined in the phase 2 study. Sensitivity, specificity, accuracy, likelihood ratios, and the DOR measured in our DAgS-ELISA suggest that TpN17 has significant diagnostic potential, although indirect ELISA exhibited higher overall performance.

To enhance the clinical applicability of DAgS-ELISA for syphilis diagnosis, future research should include a more diverse and extensive sample panel to ensure consistent performance across different populations and clinical scenarios. Additionally, evaluating alternative antigenic targets may further refine assay specificity and sensitivity, solidifying DAgS-ELISA as a valuable tool for syphilis diagnostics.

## 6 Limitations

The primary limitation of this study was the lack representation of key syphilis-affected subpopulations, including pregnant women, congenital syphilis cases, and individuals with tertiary syphilis. The exclusion of these groups may limit the generalizability of our findings across all disease stages and clinical presentations. Additionally, the overall sample size was smaller than those typically used in other indirect ELISA studies, which may affect the statistical power and robustness of our conclusions. Another challenge was the difficulty in obtaining well-characterized serological samples for cross-reactivity analysis. The limited number of samples, particularly the absence of *Leptospira*-positive specimens, restricted our ability to comprehensively assess potential cross-reactivity. Furthermore, the study relies on samples from a single geographic region, which may limit the generalizability of findings to populations with different genetic backgrounds or syphilis epidemiology. Despite these limitations, the optimization of DAgS-ELISA for syphilis diagnosis using TpN17 remains promising. Future studies should aim to expand sample diversity and further refine assay conditions to maximize its clinical utility.

**Issue**: The study relies on samples from a single geographic region, which may limit the generalizability of findings to populations with different genetic backgrounds or syphilis epidemiology.

**Suggestion**: Conducting external validation studies with samples from different geographic regions, particularly areas with high syphilis prevalence, would enhance the applicability of TpN17-based DAgS-ELISA.

## Data Availability

The raw data supporting the conclusions of this article will be made available by the authors, without undue reservation.

## Footnotes

^1^ https://www.openepi.com
